# Predictive factors for missed adenoma on repeat colonoscopy in patients with suboptimal bowel preparation on initial colonoscopy: A KASID multicenter study

**DOI:** 10.1371/journal.pone.0195709

**Published:** 2018-04-26

**Authors:** Ji Young Chang, Chang Mo Moon, Hyun Jung Lee, Hyo-Joon Yang, Yunho Jung, Sang Wook Kim, Sung-Ae Jung, Jeong-Sik Byeon

**Affiliations:** 1 Department of Internal Medicine, College of Medicine, Ewha Womans University, Seoul, Republic of Korea; 2 Tissue Injury Defense Research Center, Ewha Womans University, Seoul, Republic of Korea; 3 Department of Internal Medicine and Institute of Gastroenterology, Yonsei University College of Medicine, Seoul, Republic of Korea; 4 Division of Gastroenterology, Department of Internal Medicine and Gastrointestinal Cancer Center, Kangbuk Samsung Hospital, Sungkyunkwan University School of Medicine, Seoul, Republic of Korea; 5 Department of Internal Medicine, Division of Gastroenterology, Soonchunhyang University College of Medicine, Cheonan, Republic of Korea; 6 Department of Internal Medicine, Chonbuk National University College of Medicine, Jeonju, Republic of Korea; 7 Department of Gastroenterology, Asan Medical Center, University of Ulsan College of Medicine, Seoul, Republic of Korea; University of Munich, GERMANY

## Abstract

Suboptimal bowel preparation can result in missed colorectal adenoma that can evolve into interval colorectal cancer. This study aims to identify the predictive factors associated with missed adenoma on repeat colonoscopy in patients with suboptimal bowel preparation at initial colonoscopy. A total of 441 patients with suboptimal bowel preparation on initial colonoscopy and who had repeat colonoscopy within two years were included from 2007 to 2014 in six tertiary hospitals. Suboptimal bowel preparation was defined as ‘poor’ according to the Aronchick scale or a score ≤ 1 in at least one segment or total score < 6 according to the Boston bowel preparation scale. Of 441 patients, mean age at initial colonoscopy was 59.1 years, and 69.2% patients were male. The mean interval from initial to repeat colonoscopy was 14.1 months. The per-patient adenoma miss rate (AMR) was 42.4% for any adenoma and 5.4% for advanced adenoma. When the association between baseline clinical characteristics and missed lesions on repeat colonoscopy was analyzed, dyslipidemia (odds ratio [OR], 5.19; 95% confidence interval [CI], 1.14–23.66; *P* = 0.034), and high-risk adenoma (OR, 4.45; 95% CI, 1.12–17.68; *P* = 0.034) on initial colonoscopy were independent risk factors for missed advanced adenoma. In patients with suboptimal bowel preparation, dyslipidemia and high-risk adenoma on initial colonoscopy were independently predictive of missed advanced adenoma on repeat colonoscopy.

## Introduction

Colonoscopy is an evidence-based modality that can reduce the incidence of colorectal cancer (CRC) and cancer-related mortality [1,2]. For effective colonoscopy, adequate bowel preparation is essential and crucial. Adequate bowel preparation is closely related to colorectal adenoma (CRA) detection and safety during the procedure [3]. However, in real practice, suboptimal bowel preparation occurs in 25–30% of all colonoscopies [4,5]. Suboptimal bowel preparation may not only attenuate the protective role of colonoscopy, but also leads to additional examinations. This can increase medical cost [6,7] or the number of procedure-related complications [7]. In addition, suboptimal bowel preparation is a major possible cause of interval CRC [8]. Risk of missed CRA is two to three times higher with suboptimal bowel preparation compared to cases with excellent bowel preparation [7].

The recent 2012 US Multi-Society Task Force on Colorectal Cancer guideline for colonoscopy surveillance mentioned poor preparation for the first time and recommends repeat examination within 1 year in most cases of poor bowel preparation [9]. This guideline suggests that the goal of adequate preparation is visualization of polyps larger than 5 mm. However, the definition terms “poor” and “in most cases” in this guideline are subjective versus definitive and this recommendation lacks sufficient evidence. Most previous studies regarding suboptimal bowel preparation have concluded that suboptimal bowel preparation is associated with missed CRA [7,10,11]. However, there have not been any studies to evaluate clinical risk factors that predict missed colorectal lesions in patients with suboptimal bowel preparation.

The aim of this study is to identify the clinical predictive factors associated with missed CRA on repeat colonoscopy in patients with suboptimal bowel preparation on initial colonoscopy.

## Materials and methods

### Study subjects

In this multicenter, retrospective study, the study population consisted of adult subjects aged 30 to 75 years old who showed suboptimal preparation on their initial colonoscopy and underwent repeat colonoscopy within two years from January 2007 and December 2014 (n = 809). They underwent initial colonoscopy for screening or surveillance of CRC, diagnosis of presenting symptoms, or therapy of known colon polyp. Suboptimal bowel preparation of initial colonoscopy was ‘poor’ or ‘inadequate’ according to the Aronchick Bowel Preparation Scale (ABPS) or ≤ 1 in at least one segment or total score < 6 according to the Boston Bowel Preparation Scale (BBPS). Initial and repeat colonoscopy was completely examined and interval between these two examination was less than 2 years. The clinical data of these subjects were collected from six tertiary medical institutions (Ewha Womans University School of Medicine, Yonsei University College of Medicine, Sungkyunkwan University School of Medicine, Soonchunhyang University College of Medicine, Chonbuk National University College of Medicine, and University of Ulsan College of Medicine) in republic of Korea.

Subjects were excluded by the following criteria: (1) failure of cecal intubation on initial colonoscopy; (2) bowel resection history; (3) incomplete removal of polyps found on initial colonoscopy; (4) known or newly diagnosed inflammatory bowel disease (IBD) or CRC; (5) inadequate bowel preparation of repeat colonoscopy; (6) insufficient medical records. For patients who were referred to one of six hospitals for polyp resection, only cases in which lesions detected at the referring hospital could be definitely recognized on initial colonoscopy by their location, shape, and size were included. After excluding individuals with aforementioned exclusion criteria, a total of 441 subjects were included in this study.

All colonoscopies were done with standard colonoscopes (CF Q240, CF Q260, CF H260; Olympus Optical Co., Ltd., Tokyo, Japan) with EVIS LUCERA system (Olympus, Tokyo, Japan).

### Assessment of bowel preparation quality

An endoscopist assessed bowel preparation quality during examinations using BBPS or ABPS. BBPS assesses bowel preparation using the following 4-point scale for three colon segments (right, transverse, left). Unprepared colon with an unapparent entire mucosa because of solid non-removable stool is scored as 0. Poor visualization of some portion of mucosa due to residual stool and/or opaque liquid and staining is scored as 1. Good visualization of most mucosa with a minor amount of small stool fragments and/or opaque liquid and residual staining is scored as 2, and perfect visualization of the entire mucosa is scored as 3. The right colon includes large bowel from the cecum to hepatic flexure, the transverse colon is from the hepatic to splenic flexures, and the left colon is defined as the splenic flexure to the rectum. Total BBPS score is the sum of scores at each segment, ranging from 0 to 9 [12,13]. ABPS assesses the bowel preparation quality of the entire colon using 5 scales; examinations that require repeat colonoscopy are ‘inadequate’, and visualization of less than 90% of the mucosal surface due to semisolid stool that cannot be suctioned or washed away is referred to as ‘poor’. ‘Fair’ is when greater than 90% of the colon surface is seen with some semisolid stool that cannot be suctioned or washed out, whereas ‘good’ is defined if there is only a large volume of clear liquid. ‘Excellent’ describes examinations in which greater than 95% of the mucosal surface are seen or only a small volume of clear liquid is present [14]. In our study, suboptimal bowel preparation was defined as ‘poor’ or ‘inadequate’ according to ABPS and a score ≤ 1 in at least one segment or total score < 6 according to BBPS.

### Study outcomes

During initial and repeat examinations, all detected polyps were resected by forcep removal, endoscopic mucosal resection, or snare polypectomy. Advanced adenoma was defined as tubular adenoma greater than 10 mm in diameter, any adenoma containing villous histological features, or adenoma with high-grade dysplasia [15]. The low-risk adenoma group was defined as 1 or 2 tubular adenomas without features of advanced adenomas, whereas the high-risk adenoma group included advanced adenomas or more than 3 tubular adenomas without features of advanced adenoma. Adenomas found on repeat colonoscopy but not on initial colonoscopy were defined as missed adenomas. The per-patient adenoma miss rate (AMR) was calculated as (number of patient with missed adenoma) / (total number of patients) [16,17]. The per-adenoma AMR was defined as (number of missed adenomas) / (total number of adenomas found at both initial and repeat examinations) [16,17].

The following demographic and clinical data were reviewed through medical records to analyze risk factors associated with missed adenoma: demographic information, body mass index (BMI), smoking habits and alcohol consumption, a family history of CRC, a history of colon polyps, a history of abdominal surgery, comorbidities, indication for initial colonoscopy, bowel preparation materials and methods, patient admission status on initial colonoscopy, participation of trainee, withdrawal time, initial colonoscopic findings, and interval between initial and repeat colonoscopy.

### Statistical analysis

All statistical analyses were carried out using SPSS program, version 22.0. To evaluate the prevalence of missed adenoma according to colon location in the per–patient method, Cochran’s Q test (MedCalc, version 11.5.1.0) and Fisher’s exact test were used. In univariate analyses, Student *t-*test was used for continuous variables and the chi-square test, Fisher’s exact test, or Linear-by-Linear association was used for categorical variables. Multivariate logistic regression analysis was used to identify independent predictive factors for any or advanced missed adenomas. *P* values of < 0.05 were considered statistically significant.

### Ethics statement

This study was approved by the Institutional Review Board of Ewha Womans University Mokdong Hospital (IRB number; 2015-12-035), and written consent was waived because of the retrospective design of the study.

## Results

### Baseline characteristics

Baseline characteristics of study subjects and initial colonoscopy are shown in Table 1. The mean age of the study population was 59.1±11.0 years, and 69.2% were male. Mean BMI was 24.4 kg/m^2^, and 30.8% of patients had a history of colon polyp. Regarding indication for colonoscopy, screening or surveillance was noted in 362 (82.1%) patients, diagnosis of specific symptoms was cited in 58 (13.2%) patients, and therapeutic purposes were noted in 21 (4.8%) patients. A total of 239 (54.2%) cases were assessed by ABPS, and 202 (45.8%) cases were assessed by BBPS. For bowel preparation, 246 (55.8%) patients were prescribed a split dosing regimen and 357 (81.0%) patients were out-patients. Mean withdrawal time during initial colonoscopy was 15.7±11.9 minutes. The characteristics of repeat colonoscopy are summarized in S1 Table. The mean interval from initial to repeat colonoscopic examination was 14.1 months (median 13.1 months).

**Table 1:** Baseline characteristics of the study subjects and initial colonoscopy.

N = 441	
Age at initial colonoscopy (years), mean ± SD	59.1 ± 11.0
Male sex (%)	305 (69.2)
BMI (Kg/m^2^), mean ± SD	24.4 ± 3.1
Smoking habit (%)	
No	277 (62.8)
Ex-smoker	74 (16.8)
Current	90 (20.4)
Alcohol consumption (%)	
No	225 (51.0)
Social	181 (41.0)
Heavy	35 (7.9)
Family history of colorectal cancer (%)	
No	428 (97.1)
Yes	13 (2.9)
History of colon polyp (%)	
No	305 (69.2)
Yes	136 (30.8)
History of abdomen surgery (%)	
No	366 (83.0)
Low-risk^a^	36 (8.2)
High-risk^b^	39 (8.8)
Comorbidity (%)	
Hypertension	142 (32.2)
Diabetes mellitus	80 (18.1)
Dyslipidemia	40 (9.1)
Arterial thromboembolic disease^c^	21 (4.8)
Indication for colonoscopy (%)	
Screen or Surveillance	362 (82.1)
Diagnostic purpose	58 (13.2)
Bowel habit change	21 (4.8)
Abdominal pain	18 (4.1)
Hematochezia	10 (2.3)
Iron deficiency anemia	5 (1.1)
Positive stool occult blood	3 (0.7)
Weight loss	1 (0.2)
Therapeutic purpose	21 (4.8)
Bowel preparation scale	
ABPS	239 (54.2)
BBPS	202 (45.8)
Bowel preparation material, n (%)	
4L PEG	320 (72.6)
Sodium picosulfate + magnesium oxide	58 (13.2)
2L PEG + ascorbic acid	54 (12.2)
Others^d^	9 (2.0)
Bowel preparation method, n (%)	
Split	246 (55.8)
Same day	195 (44.2)
Out-patient, n (%)	
No	84 (19.0)
Yes	357 (81.0)
Endoscopists’ experience, n (%)	
Trainee	197 (44.7)
Expert	244 (55.4)
Withdrawal time, mean ± SD (min)	15.7 ± 11.9
Colonoscopy finding, n (%)	
No adenoma	187 (42.4)
Low-risk adenoma^e^	142 (32.2)
High-risk adenoma^f^	112 (25.4)
Interval, mean ± SD (months)	14.1 ± 6.0

SD, standard deviation; BMI, Body mass index; ABPS, Aronchick bowel preparation scale; BBPS, Boston bowel preparation scale; PEG, polyethylene glycol.

^a^Abdominal surgery with low-risk of incomplete colonoscopic insertion included appendectomy, cholecystectomy, hernia repair.

^b^Abdominal surgery with high-risk of incomplete colonoscopic insertion included extensive abdominal operation, pelvic surgery, gynecologic surgery.

^c^Arterial thromboembolic disease included ischemic heart disease or stroke.

^d^Others included as following, 2L PEG ± bisacodyl, 3L PEG, 4L PEG + bisacodyl, 6L PEG, 8L PEG, 3L PEG + ascorbic acid, Macrogol solution + bisacodyl, sodium phosphate.

^e^Low-risk adenoma was defined 1 or 2 adenomas without advanced adenoma feature.

^f^High-risk adenoma included advanced adenoma or more than equal to 3 adenomas.

### Adenoma miss rates

**Fig 1:**
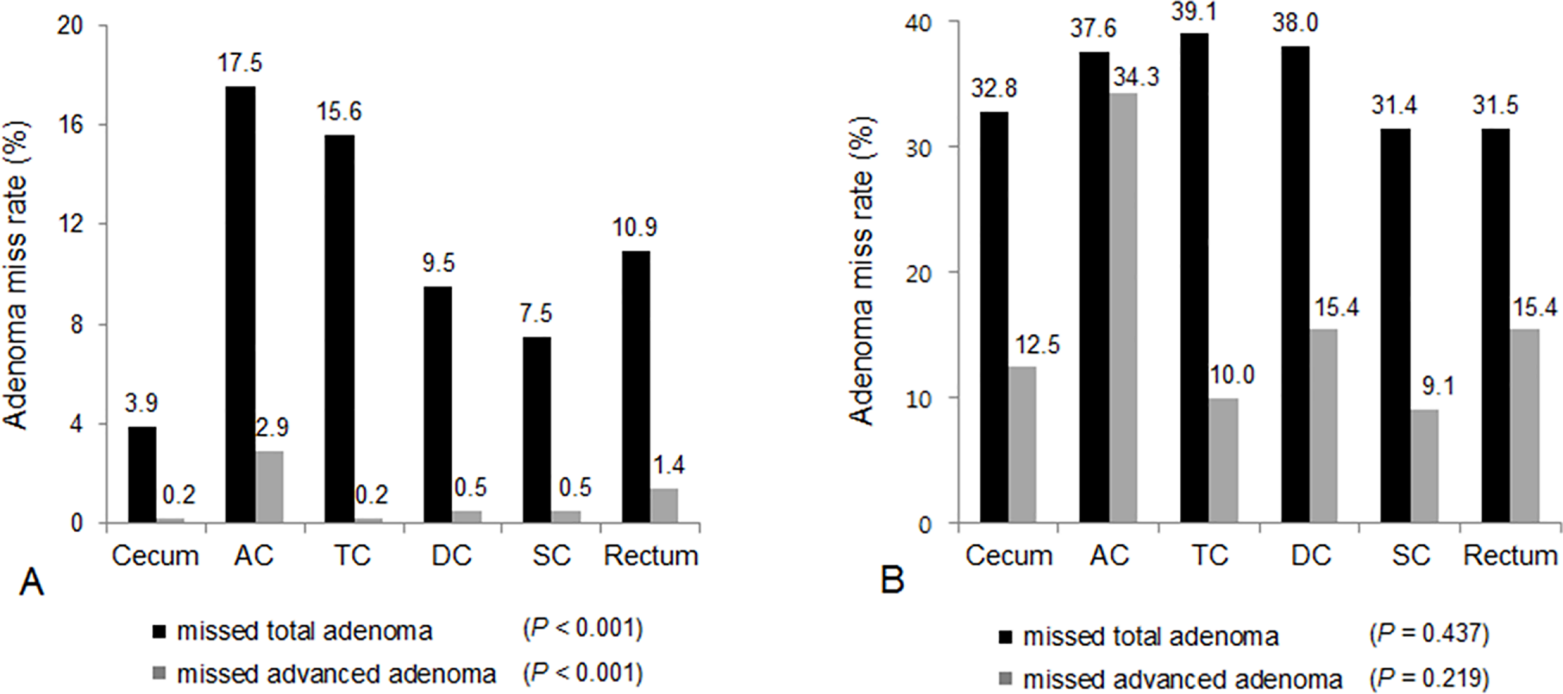
Adenoma miss rates (AMR) according to colonic location. Per-patient AMR according to colonic segment (Fig 1A). Proportion of missed total and missed advanced adenomas was significantly different according to location (*P* < 0.001). Per-adenoma AMR according to colonic segment (Fig 1B). There was no significant difference in proportion of missed total adenoma (*P* = 0.437). The AMR for advanced adenoma was 34.3% at ascending colon, twice as high as the AMR in other segments, but this difference was not statistically significant (*P* = 0.219). AC, ascending colon; TC, transverse colon; DC, descending colon; SC, sigmoid colon.

Among 441 patients, missed adenomas were detected in 187 patients, resulting in per-patient AMR for any adenoma of 42.4%. Among a total of 1,079 adenomas, 386 adenomas were missed on initial colonoscopy, representing 35.8% per-adenoma AMR. A total of 127 advanced adenomas were found at both initial and repeat colonoscopy in 441 patients. Twenty-four advanced adenomas were missed on initial colonoscopy in 24 patients. Thus, the per-patient AMR was 5.4% and per-adenoma AMR was 18.9% for advanced adenoma, respectively (Table 2). When missed adenomas were analyzed according to location (Fig 1), missed total adenomas were most frequently at the ascending colon (17.5%), followed by the transverse colon (15.6%) and rectum (10.9%). A majority of missed advanced adenomas were found at the ascending colon (2.9%) and rectum (1.4%) in per-patient analysis (Fig 1A). The proportion of missed total and missed advanced adenomas was significantly different according to location (*P* < 0.001). In per-adenoma analysis (Fig 1B), the location of missed total adenomas in descending order was transverse colon (39.1%), descending colon (38.0%) and ascending colon (37.6%), with no significant difference between sites (*P* = 0.437). AMR for advanced adenoma was 34.3% at the ascending colon, twice as high as the AMR in other segments (15.4% at descending colon, 15.4% at rectum, 12.5% at cecum, 10.0% at transverse colon, and 9.1% at sigmoid colon). However, this difference was not statistically significant (*P* = 0.219).

**Table 2:** Adenoma miss rate for any adenoma and advanced adenoma.

	Any adenoma	Advanced adenoma
Per-patient AMR, %	42.4 (187/441)	5.4 (24/441)
Per-adenoma AMR, %	35.8 (386/1,079)	18.9 (24/127)

AMR, adenoma miss rate.

To evaluate differences in AMR according to the grade of bowel preparation quality on initial colonoscopy, AMR according to total BBPS score was analyzed in 202 (45.8%) cases in which initial colonoscopy was assessed by BBPS scale. When the AMR of each colon portion was analyzed according to the segmental BBPS score, there was no significant difference in AMR for any adenoma and advanced adenoma based on BBPS segment score at all three colon regions (S2 Table).

### Predictive factors for missed adenoma on repeat colonoscopy

To identify predictive factors for missed adenoma, the association of baseline clinical characteristics on initial colonoscopy with missed CRA on repeat colonoscopy was analyzed. Age at initial colonoscopy (*P* < 0.001), BMI (*P* = 0.026), alcohol (*P* = 0.028), history of colon polyps (*P* < 0.001), hypertension (*P* < 0.001), diabetes mellitus (*P* = 0.023), dyslipidemia (*P* = 0.007), arterial thromboembolic disease (*P* = 0.001), initial colonoscopy findings (*P* < 0.001), and withdrawal time (*P* = 0.018) were significantly associated with any missed adenoma on repeat colonoscopy in univariate analysis. In multivariate analysis including all variables, BMI (BMI ≥ 25 kg/m^2^) (odds ratio [OR], 1.55; 95% confidence interval [CI], 1.00–2.40; *P* = 0.049), a history of colon polyps (OR, 1.98; 95% CI, 1.23–3.19; *P* = 0.005), arterial thromboembolic disease (OR, 3.46; 95% CI,1.09–11.02; *P* = 0.036), and both low-risk adenoma (OR, 2.03; 95% CI, 1.23–3.36; *P* = 0.006) and high-risk adenoma (OR, 4.19; 95% CI, 2.36–7.44; *P* < 0.001) on initial colonoscopy were independent predictors for any missed adenoma on repeat examination (Table 3).

**Table 3:** Clinical factors at initial colonoscopy predictive of any missed adenoma of repeat colonoscopy.

	Univariate			Multivariate		
	No adenoma	Any adenoma at repeat colonoscopy	*P*	OR	95% CI	*P*
Age at initial colonoscopy			< 0.001			
< 60	147 (66.8)	73 (33.2)		(ref)		
≥ 60	107 (48.4)	114 (51.6)		1.53	0.97–2.41	0.065
Sex			0.110			
Female	86 (63.2)	50 (36.8)		(ref)		
Male	168 (55.1)	137 (44.9)		0.99	0.58–1.69	0.970
BMI			0.026			
< 25	160 (62.0)	98 (38.0)		(ref)		
≥ 25	94 (51.4)	89 (48.6)		1.55	1.00–2.40	0.049
Smoking			0.565			
Never	165 (59.6)	112 (40.4)		(ref)		
Ex-smoker	40 (54.1)	34 (45.9)		0.71	0.36–1.41	0.330
Current	49 (54.4)	41 (45.6)		1.00	0.54–1.87	0.992
Alcohol intake			0.028			
No	141 (62.7)	84 (37.3)		(ref)		
Social & heavy	113 (52.3)	103 (47.7)		1.26	0.75–2.11	0.387
Family history of CRC			0.156			
No	249 (58.2)	179 (41.8)		(ref)		
Yes	5 (38.5)	8 (61.5)		2.38	0.69–8.17	0.168
History of colon polyp			< 0.001			
No	193 (63.3)	112 (36.7)		(ref)	-	
Yes	61 (44.9)	75 (55.1)		1.98	1.23–3.19	0.005
Hypertension			< 0.001			
No	191 (63.9)	108 (36.1)		(ref)		
Yes	63 (44.4)	79 (55.6)		1.52	0.93–2.49	0.097
Diabetes mellitus			0.023			
No	217 (60.1)	144 (39.9)		(ref)		
Yes	37 (46.2)	43 (53.8)		1.09	0.60–1.96	0.777
Dyslipidemia			0.007			
No	239 (59.6)	162 (40.4)		(ref)		
Yes	15 (37.5)	25 (62.5)		1.94	0.88–4.29	0.102
Arterial thromboembolic disease^a^			0.001			
No	249 (59.3)	171 (40.7)		(ref)		
Yes	5 (23.8)	16 (76.2)		3.46	1.09–11.02	0.036
Initial colonoscopy finding			< 0.001			
No adenoma	134 (71.7)	53 (28.3)		(ref)		
Low-risk adenoma^b^	78 (54.9)	64 (45.1)		2.03	1.23–3.36	0.006
High-risk adenoma^c^	42 (37.5)	70 (62.5)		4.19	2.36–7.44	< 0.001
Interval^d^			0.395			
< 13 month	120 (55.6)	96 (44.4)		(ref)		
≥ 13 month	134 (59.6)	91 (40.4)		0.95	0.61–1.46	0.806
Out-patients			0.691			
No	50 (59.5)	34 (40.5)		(ref)		
Yes	204 (57.1)	153 (42.9)		1.17	0.65–2.12	0.603
Endoscopists’ experience			0.623			
Expert	138 (56.6)	106 (43.4)		(ref)		
Trainee	116 (58.9)	81 (41.1)		1.36	0.87–2.12	0.178
Indication for initial colonoscopy			0.900			
Screen or surveillance	208 (57.5)	154 (42.5)		(ref)		
Diagnostic or therapeutic	46 (58.2)	33 (41.8)		1.05	0.58–1.90	0.881
Withdrawal time			0.018			
< 6 minutes	48 (70.6)	20 (29.4)		(ref)		
≥ 6 minutes	206 (55.2)	167 (44.8)		1.28	0.67–2.45	0.458

BMI, body mass index; CRC, colorectal cancer; OR, odds ratio; CI, confidence interval.

^a^Arterial thromboembolic disease included ischemic heart disease or stroke.

^b^Low-risk adenoma group was defined as 1 or 2 tubular adenoma without feature of advanced adenoma.

^c^High-risk adenoma group included advanced adenomas or more than 3 tubular adenomas without the feature of advanced adenoma.

^d^Interval means the duration from the initial to repeat colonoscopy, and the median was 13 months.

Dyslipidemia (OR, 5.19; 95% CI, 1.14–23.66; *P* = 0.034), and high-risk adenoma (OR, 4.45; 95% CI, 1.12–17.68; *P* = 0.034) on initial colonoscopy were independent predictive factors for missed advanced adenoma on repeat colonoscopy (Table 4). Additionally, older age (≥ 60 years) (OR, 2.78; 95% CI, 1.30–5.94; *P* = 0.009), hypertension (OR, 2.32; 95% CI, 1.13–4.78; *P* = 0.022) and high-risk adenoma on initial colonoscopy (OR, 5.65; 95% CI, 2.40–13.26; *P* < 0.001) were independently predictive of high-risk adenoma on repeat colonoscopy (S3 Table).

**Table 4:** Clinical factors at initial colonoscopy predictive of missed advanced adenoma of repeat colonoscopy.

	Univariate			Multivariate		
	No advanced adenoma at repeat colonoscopy	Advanced adenoma at repeat colonoscopy	*P*	OR	95% CI	*P*
Age at initial colonoscopy			0.055			
< 60	215 (97.7)	5 (2.3)		(ref)		
≥ 60	208 (94.1)	13 (5.9)		1.68	0.50–5.63	0.401
Sex			0.774			
Female	131 (96.3)	5 (3.7)		(ref)		
Male	292 (95.7)	13 (4.3)		1.03	0.30–3.62	0.960
BMI			0.796			
< 25	248 (96.1)	10 (3.9)		(ref)		
≥ 25	175 (95.6)	8 (4.4)		0.97	0.34–2.76	0.958
Smoking			0.832			
Never	264 (95.3)	13 (4.7)		(ref)		
Ex-smoker	72 (97.3)	2 (2.7)		0.26	0.04–1.64	0.152
Current	87 (96.7)	3 (3.3)		0.34	0.07–1.71	0.191
Alcohol intake			0.293			
No	218 (96.9)	7 (3.1)		(ref)		
Social & heavy	205 (94.9)	11 (5.1)		1.77	0.50–6.22	0.375
Family history of CRC			0.423			
No	411 (96.0)	17 (4.0)		(ref)		
Yes	12 (92.3)	1 (7.7)		2.84	0.28–28.36	0.375
History of colon polyp			0.072			
No	296 (97.0)	9 (3.0)		(ref)		
Yes	127 (93.4)	9 (6.6)		2.74	0.92–8.16	0.071
Hypertension			0.099			
No	290 (97.0)	9 (3.0)		(ref)		
Yes	133 (93.7)	9 (6.3)		1.62	0.50–5.28	0.419
Diabetes mellitus			> 0.999			
No	346 (95.8)	15 (4.2)		(ref)		
Yes	77 (96.3)	3 (3.7)		0.27	0.05–1.30	0.102
Dyslipidemia			0.070			
No	387 (96.5)	14 (3.5)		(ref)		
Yes	36 (90.0)	4 (10.0)		5.19	1.14–23.66	0.034
Arterial thromboembolic disease^a^			0.592			
No	403 (96.0)	17 (4.0)		(ref)		
Yes	20 (95.2)	1 (4.8)		0.82	0.08–8.19	0.865
Initial colonoscopy finding			0.019			
No adenoma	183 (97.9)	4 (2.1)		(ref)		
Low-risk adenoma^b^	138 (97.2)	4 (2.8)		1.31	0.30–5.84	0.722
High-risk adenoma^c^	102 (91.1)	10 (8.9)		4.45	1.12–17.68	0.034
Interval^d^			0.569			
< 13 month	206 (95.4)	10 (4.6)		(ref)		
≥ 13 month	217 (96.4)	8 (3.6)		1.21	0.41–3.53	0.733
Out-patients			0.759			
No	80 (95.2)	4 (4.8)		(ref)		
Yes	343 (96.1)	14 (3.9)		0.44	0.11–1.83	0.259
Endoscopists’ experience			0.614			
Expert	233 (95.5)	11 (4.5)		(ref)		
Trainee	190 (96.4)	7 (3.6)		1.10	0.36–3.39	0.866
Indication for initial colonoscopy			0.752			
Screen or surveillance	346 (95.6)	16 (4.4)		(ref)		
Diagnostic or therapeutic	77 (97.5)	2 (2.5)		0.34	0.06–2.07	0.241
Withdrawal time			0.331			
< 6 minutes	67 (98.5)	1 (1.5)		(ref)		
≥ 6 minutes	356 (95.4)	17 (4.6)		2.37	0.25–22.01	0.449

BMI, body mass index; CRC, colorectal cancer; OR, odds ratio; CI, confidence interval.

^a^Arterial thromboembolic disease included ischemic heart disease or stroke.

^b^Low-risk adenoma group was defined as 1 or 2 tubular adenoma without feature of advanced adenoma.

^c^High-risk adenoma group included advanced adenomas or more than 3 tubular adenomas without the feature of advanced adenoma.

^d^Interval means the duration from the initial to repeat colonoscopy, and the median was 13 months.

## Discussion

This study identified clinical predictors associated with missed CRA on repeat colonoscopy in patients with suboptimal bowel preparation. This study demonstrated that the per-patient AMR was 42.4% for any adenoma and 5.4% for advanced adenoma. Several previous studies showed the negative impact of suboptimal bowel preparation on missing CRA by reporting per-patient AMR for any adenoma and advanced adenoma as 33.8–47% and 18.0–37%, respectively [7,10]. When AMR was analyzed by the per-adenoma method, the rate was 40 to 47.9% for any adenoma and 27 to 58% for advanced adenoma [4,7,10]. The results of our study are comparable to other studies presenting the AMR for any adenoma as 42.4% in per-patient analysis and 35.5% in per-adenoma analysis. However, our study revealed a lower AMR for missed advanced adenoma (5.4% in per-patient analysis and 18.9% per-adenoma analysis) compared to previous studies. This difference might be explained by the difference in prevalence, location, or histologic characteristics of CRAs according to geographic or racial differences. The Korean population had higher risk of distal adenoma and large adenoma compared to the U.S population [18]. The AMR is also influenced by various factors related to endoscopists, instruments, and surveillance interval, which are associated with the quality of colonoscopy [19–21]. Thus, large-scale studies including multiple racial populations are needed to explain different AMR values.

Our study showed that the per-patient AMR for both any adenoma (*P* < 0.001) and advanced adenoma (*P* < 0.001) was significantly higher in the ascending colon compared with other segments. To our knowledge, only a few studies have evaluated AMR according to colon location. Chokshi et al [10] reported 64.8% of total missed adenomas and 80.0% of missed advanced adenomas were found in the proximal colon. Singhal et al [22] reported 66.6% of missed adenomas were located in the right colon. This can be explained by tumor biology or morphology [23,24] and anatomical structure, with prominent folds leading to lower adenoma recognition even with adequate bowel preparation [25].

We also evaluated differences in AMR according to grade of bowel preparation quality on initial colonoscopy. There was no significant difference in AMR for any adenoma and advanced adenoma between different groups according to the BBPS segment score. This result is different from that of the prior study [26]. Clark et al [26] suggest BBPS segment score of 1 as a threshold for inadequate preparation requiring early repeat examination because BBPS segment score less than 1 had a significantly higher rate of AMR and changed the recommend surveillance interval in more than 40% of patients. We considered several reasons for this result. First, the relatively small sample size of our study could cause this statistical insignificance. Second, according to some inclusion criteria of our study such as the completion of initial colonoscopy, this study may have excluded some patients with quite poor bowel preparation, which could have resulted in ‘filtering’ the subjects showing relatively similar bowel preparation grades.

Our study found that dyslipidemia, and high-risk adenoma on the initial colonoscopy are independent predictors for missed advanced adenoma of repeat colonoscopy. Although there have been no prior studies evaluating the predictive factors of missed CRA in patients with initial suboptimal bowel preparation, these factors can be explained by studies investigating predictors for metachronous or missed colorectal neoplasm in routine surveillance. Patient age [27], male sex [28], increased number [27–30], size [27–29], dysplasia [30], and villous histologic component [28] of adenoma on initial colonoscopy have been identified as risk factors for metachronous neoplasia. Dyslipidemia, a component of metabolic syndrome, is a risk factor of metachronous CRA as well as CRA prevalence [31]. Dyslipidemia causes insulin resistance and tumorigenesis [32]. For factors associated with missed adenoma, sessile shape, location, size, and number were reported in tandem colonoscopic studies [16,33]. High-risk adenoma, which implies increase an in number and size, dysplasia, and villous histologic component were commonly associated factors for missed advanced adenoma or metachronous neoplasm. Some predictors for missed CRA in patients with suboptimal preparation, such as history of colon polyps and CRA on initial colonoscopy, are in line with those for missed or metachronous CRA in routine surveillance.

This study has several limitations. First, its retrospective design may cause selection or recall biases. Also, interobserver or intraobserver bias may exist in the assessment of bowel preparation. However, there are several difficulties in conducting a prospective study in this setting. Allocation of study subjects to a group with suboptimal bowel preparation could give rise to ethical problems, and recruiting a sufficient sample size would take long-term study period. Second, we assumed that newly detected CRA in the repeat examination was a missed lesion, but these lesions may be interpreted as either missed CRA or newly developed metachronous CRA. To minimize this controversy, we only included study subjects whose repeat colonoscopy was conducted within 2 years. Third, after excluding subjects with predictive factors for CRA such as history of IBD or CRC, there are still heterogeneous indications for colonoscopy in this study. A recent study demonstrated that the ADR differed depending on the indication of colonoscopy, yielding an overall ADR as 22.9% in patients undergoing screening colonoscopy, 36.1% in surveillance colonoscopy, and from 12% to 30% in diagnostic colonoscopy for gastrointestinal symptoms [34]. Different indications in study subjects can influence results in our study. Fourth, as the present study was conducted in a retrospective manner, not all repeat colonoscopies were done by the same endoscopist, who had performed the initial examination. However, since this study was conducted at tertiary university hospitals certified for high-level quality-control for colonoscopy, the ADR of each endoscopist, withdrawal time, or bowel preparation were under comprehensive control. Thus, discrepancies of endoscopists between initial and repeat colonoscopy would not have affected the outcome in this study. Additionally, this study did not analyze AMR on repeat colonoscopy of patients with inadequate bowel preparation compared with those with adequate bowel preparation. To evaluate how much inadequate bowel preparation can influence on AMR, comparison group will be necessary for the analysis. Lastly, although we included the initial colonoscopy finding as a variable for multivariate analysis based on the size, number, and histology, we could not analyze the influence of the shape of adenoma on AMR. Since there have been conflicting results regarding the association between AMR and polyp shape [17,35,36], further studies are needed. However, the main aim of this study is to identify the predictive factors associated with missed adenoma on repeat colonoscopy in patients with suboptimal bowel preparation at initial colonoscopy. If colonoscopy can reach cecum despite the poor bowel preparation, physicians have to consider a number of factors, including how soon the next colonoscopy should be performed and what clinical factors can affect the prevalence of missed adenoma at repeat colonoscopy. These situations are often encountered in the real clinical practice and we focused this issue. However, further studies comparing the AMR between subject groups according to the quality of bowel preparation are necessary to verify the negative effect of poor bowel preparation.

Despite these limitations, this is a large-scale study with detailed analysis of baseline characteristics of study subjects and colonoscopy. Our study was conducted at six tertiary university hospitals certified with high-level quality control for colonoscopy. A large number of patients with suboptimal bowel preparation on initial colonoscopy and reliable repeat examinations performed at the same institution were studied.

## Conclusion

In conclusion, in patients with suboptimal bowel preparation on initial colonoscopy, dyslipidemia and high-risk adenoma on initial colonoscopy were significant predictors for missed advanced adenoma on repeat colonoscopy. Accordingly, we suggest that special attention is needed in patients with such predictive factors when deciding on colonoscopic surveillance strategy.

## Supporting information

S1 Table Characteristics of the repeat colonoscopy. (DOCX)

S2 Table Adenoma miss rates according to the BBPS segment score at initial colonoscopy (N = 202). (DOCX)

S3 Table Clinical factors at initial colonoscopy predictive of high-risk adenoma of repeat colonoscopy. (DOCX)
