# Supplementary material for: Predictive factors for missed adenoma on repeat colonoscopy in patients with suboptimal bowel preparation on initial colonoscopy: A KASID multicenter study
Source: PLoS One. 2018 Apr 26;13(4):e0195709. doi: 10.1371/journal.pone.0195709 (PMC5919514; doi:10.1371/journal.pone.0195709)
Supplement: S1 Table — (DOCX) [file pone.0195709.s001.docx]

**S1 Table. Characteristics of the repeat colonoscopy.**

|  | **Repeat colonoscopy** |
| --- | --- |
| Bowel preparation material, n (%) |  |
| 4L PEG | 294 (66.7) |
| Sodium picosulfate + magnesium oxide | 33 (7.5) |
| 2L PEG + ascorbic acid | 100 (22.7) |
| Others^a^ | 14 (3.1) |
| Bowel preparation method, n (%) |  |
| Split | 257 (58.3) |
| Same day | 184 (41.7) |
| Location, n (%) |  |
| Out-patient | 393(89.1) |
| In-patient | 48 (10.9) |
| Endoscopist, n (%) |  |
| Trainee | 168 (38.1) |
| Expert | 273 (61.9) |
| Withdrawal time^b^, mean ± SD (min) | 11.6 ± 8.3 |
| Colonoscopy finding, n (%) |  |
| No adenoma | 254 (57.6) |
| Low-risk adenoma^c^ | 132 (29.9) |
| High-risk adenoma^d^ | 55 (12.5) |

PEG, polyethylene glycol; SD, standard deviation

^a^Others included as following, 2L PEG ± bisacodyl, 3L PEG, 4L PEG + bisacodyl, 6L PEG, 8L PEG, 3L PEG + ascorbic acid, Macrogol solution + bisacodyl, sodium phosphate.

^b^Withdrawal time was defined as the time taken for withdrawal of colonoscope from cecum to anus.

^c^Low-risk adenoma was defined 1 or 2 adenomas without advanced adenoma feature.

^d^High-risk adenoma included advanced adenoma or more than equal to 3 adenomas.
