# Supplementary material for: Predictive factors for missed adenoma on repeat colonoscopy in patients with suboptimal bowel preparation on initial colonoscopy: A KASID multicenter study
Source: PLoS One. 2018 Apr 26;13(4):e0195709. doi: 10.1371/journal.pone.0195709 (PMC5919514; doi:10.1371/journal.pone.0195709)
Supplement: S2 Table — (DOCX) [file pone.0195709.s002.docx]

**S2 Table. Adenoma miss rates according to the BBPS segment score at initial colonoscopy (N = 202).**

|  | Adenoma miss rate | | |
| --- | --- | --- | --- |
|  | AC | TC | DC |
| Per-patient | | |  |
| Any adenoma |  |  |  |
| BBPS = 0 | 11/43 (25.6) | 0/9 (0) | 3/11 (27.3) |
| BBPS = 1 | 34/145 (23.4) | 14/63 (22.2) | 22/57 (38.6) |
| BBPS = 2 | 3/13 (23.1) | 12/104 (11.5) | 16/88 (18.2) |
| BBPS = 3 | 1/1 (100) | 2/26 (7.7) | 13/46 (28.3) |
| *P* value | 0.877 | 0.271 | 0.257 |
| Advanced adenoma |  |  |  |
| BBPS = 0 | 3/43 (7.0) | 0/9 (0) | 0/11 (0) |
| BBPS = 1 | 4/145 (2.8) | 0/63 (0) | 4/57 (7.0) |
| BBPS = 2 | 0/13 (0) | 0/104 (0) | 1/88 (1.1) |
| BBPS = 3 | 0/1 (0) | 0/26 (0) | 4/46 (8.7) |
| *P* value | 0.160 | - | 0.686 |
| Per-adenoma |  |  |  |
| Any adenoma |  |  |  |
| BBPS = 0 | 11/32 (34.8) | 0/1 (0) | 3/18 (16.7) |
| BBPS = 1 | 56/172 (32.6) | 19/58 (32.8) | 45/94 (47.9) |
| BBPS = 2 | 4/9 (44.4) | 15/48 (31.3) | 21/91 (23.1) |
| BBPS = 3 | 1/2 (50.0) | 2/10 (20) | 20/76 (26.3) |
| Advanced adenoma |  |  |  |
| BBPS = 0 | 3/6 (50.0) | 0/1 (0) | 0/0 (0) |
| BBPS = 1 | 4/14 (28.6) | 0/2 (0) | 4/15 (26.7) |
| BBPS = 2 | 0/0 (0) | 0/2 (0) | 1/17 (5.9) |
| BBPS = 3 | 0/1 (0) | 0/0 (0) | 4/21 (19.0) |

BBPS, Boston bowel preparation scale; AC, ascending colon; TC, transverse colon, DC, descending colon
