# Supplementary material for: Predictive factors for missed adenoma on repeat colonoscopy in patients with suboptimal bowel preparation on initial colonoscopy: A KASID multicenter study
Source: PLoS One. 2018 Apr 26;13(4):e0195709. doi: 10.1371/journal.pone.0195709 (PMC5919514; doi:10.1371/journal.pone.0195709)
Supplement: S3 Table — (DOCX) [file pone.0195709.s003.docx]

**S3 Table. Clinical factors at initial colonoscopy predictive of high-risk adenoma of repeat colonoscopy.**

|  | Univariate | | | Multivariate | | |
| --- | --- | --- | --- | --- | --- | --- |
|  | No high-risk adenoma at repeat colonoscopy | High-risk adenoma at repeat colonoscopy | *P* | OR | 95% CI | *P* |
| Age at initial colonoscopy |  |  | < 0.001 |  |  |  |
| < 60 | 208 (94.5) | 12 (5.5) |  | (ref) |  |  |
| ≥ 60 | 178 (80.5) | 43 (19.5) |  | 2.78 | 1.30-5.94 | 0.009 |
| Sex |  |  | 0.216 |  |  |  |
| Female | 123 (90.4) | 13 (9.6) |  | (ref) |  |  |
| Male | 263 (86.2) | 42 (13.8) |  | 0.99 | 0.42-2.29 | 0.972 |
| BMI |  |  | 0.594 |  |  |  |
| < 25 | 224 (86.8) | 34 (13.2) |  | (ref) |  |  |
| ≥ 25 | 162 (88.5) | 21 (11.5) |  | 0.73 | 0.37-1.42 | 0.349 |
| Smoking |  |  | 0.717 |  |  |  |
| Never | 245 (88.4) | 32 (11.6) |  | (ref) |  |  |
| Ex-smoker | 63 (85.1) | 11 (14.9) |  | 0.67 | 0.25-1.82 | 0.433 |
| Current | 78 (86.7) | 12 (13.3) |  | 0.91 | 0.36-2.31 | 0.843 |
| Alcohol intake |  |  | 0.020 |  |  |  |
| No | 205 (91.1) | 20 (8.9) |  | (ref) |  |  |
| Social & heavy | 181 (83.8) | 35 (16.2) |  | 1.74 | 0.77-3.91 | 0.182 |
| Family history of CRC |  |  | 0.066 |  |  |  |
| No | 377 (88.1) | 51 (11.9) |  | (ref) |  |  |
| Yes | 9 (69.2) | 4 (30.8) |  | 3.55 | 0.83-15.24 | 0.088 |
| History of colon polyp |  |  | 0.116 |  |  |  |
| No | 272 (89.2) | 33 (10.8) |  | (ref) |  |  |
| Yes | 114 (83.8) | 22 (16.2) |  | 1.35 | 0.67-2.74 | 0.400 |
| Hypertension |  |  | < 0.001 |  |  |  |
| No | 275 (92.0) | 24 (8.0) |  | (ref) |  |  |
| Yes | 111 (78.2) | 31 (21.8) |  | 2.32 | 1.13-4.78 | 0.022 |
| Diabetes mellitus |  |  | 0.024 |  |  |  |
| No | 322 (89.2) | 39 (10.8) |  | (ref) |  |  |
| Yes | 64 (80.0) | 16 (20.0) |  | 0.98 | 0.44-2.21 | 0.960 |
| Dyslipidemia |  |  | 0.021 |  |  |  |
| No | 356 (88.8) | 45 (11.2) |  | (ref) |  |  |
| Yes | 30 (75.0) | 10 (25.0) |  | 2.14 | 0.80-5.68 | 0.128 |
| Arterial thromboembolic disease^a^ |  |  | 0.163 |  |  |  |
| No | 370 (88.1) | 50 (11.9) |  | (ref) |  |  |
| Yes | 16 (76.2) | 5 (23.8) |  | 1.10 | 0.32-3.82 | 0.880 |
| Index colonoscopy findings |  |  | < 0.001 |  |  |  |
| No adenoma | 175 (93.6) | 12 (6.4) |  | (ref) |  |  |
| Low-risk adenoma^b^ | 130 (91.5) | 142 (8.5) |  | 1.43 | 0.58-3.54 | 0.441 |
| High-risk adenoma^c^ | 81 (72.3) | 31 (27.7) |  | 5.65 | 2.40-13.26 | < 0.001 |
| Interval^d^ |  |  | 0.787 |  |  |  |
| < 13 month | 190 (88.0) | 26 (12.0) |  | (ref) |  |  |
| ≥ 13 month | 196 (87.1) | 29 (12.9) |  | 1.39 | 0.71-2.70 | 0.338 |
| Out-patients |  |  | 0.861 |  |  |  |
| No | 74 (88.1) | 10 (11.9) |  | (ref) |  |  |
| Yes | 312 (87.4) | 45 (12.6) |  | 0.99 | 0.40-2.46 | 0.976 |
| Endoscopists’ experience |  |  | 0.901 |  |  |  |
| Expert | 214 (87.7) | 30 (12.3) |  | (ref) |  |  |
| Trainee | 172 (87.3) | 25 (12.7) |  | 1.60 | 0.80-3.23 | 0.184 |
| Indication for initial colonoscopy |  |  | 0.284 |  |  |  |
| Screen or surveillance | 314 (86.7) | 48 (13.3) |  | (ref) |  |  |
| Diagnostic or therapeutic | 72 (91.1) | 7 (8.9) |  | 0.46 | 0.17-1.25 | 0.128 |
| Withdrawal time |  |  | 0.074 |  |  |  |
| < 6 minutes | 64 (94.1) | 4 (5.9) |  | (ref) |  |  |
| ≥ 6 minutes | 322 (86.3) | 51 (13.7) |  | 1.25 | 0.38-4.07 | 0.711 |

BMI, body mass index; SD, standard deviation; OR, odds ratio; CI, confidence interval

^a^Arterial thromboembolic disease included ischemic heart disease or stroke.

^b^Low-risk adenoma group was defined as 1 or 2 tubular adenoma without feature of advanced adenoma

^c^High-risk adenoma group included advanced adenomas or more than 3 tubular adenomas without the feature of advanced adenoma

^d^Interval means the duration from the initial to repeat colonoscopy, and the median was 13 months.
